# circRNA THBS1 silencing inhibits the malignant biological behavior of cervical cancer cells via the regulation of miR-543/HMGB2 axis

**DOI:** 10.1515/med-2023-0709

**Published:** 2023-07-14

**Authors:** Rui Tian, Huixin Li, Songjie Ren, Shukui Li, Run Fang, Yang Liu

**Affiliations:** Gynecology Department, Shanghai Mengchao Cancer Hospital, Shanghai 201800, China; Urinary Surgery, Renhe Hospital, Baoshan District, Shanghai 200431, China; Gynaecology and Obstetrics, Lin’an District First People’s Hospital, 548 Yijin Street, Lin’an District, Hangzhou 311300, China; Urinary Surgery, Shanghai Mengchao Cancer Hospital, Shanghai 201800, China

**Keywords:** circRNA THBS1, cervical cancer cells, miR-543/HMGB2 axis

## Abstract

Circular RNA (circRNA) THBS1 has been shown to exist as an oncogene in non-small-cell lung cancer, but its role in cervical cancer is still unclear. Our experiment aimed to uncover the functions and specific mechanism of circRNA THBS1 in cervical cancer cells. Levels of circRNA THBS1 and miR-543 in cervical cancer tissues and cell lines were assessed by RT-qPCR. starBase and dual luciferase reporter gene assay were applied for investigating the correlation between miR-543 and circRNA THBS1/HMGB2. Cell proliferation and apoptosis were evaluated by MTT and flow cytometry, respectively. Furthermore, the levels of HMGB2, E-cadherin, and N-cadherin in HeLa cells were determined by RT-qPCR and western blot analysis. Our data revealed that circRNA THBS1 was significantly upregulated and miR-543 was low expressed in cervical cancer tissues and cell lines. circRNA THBS1 interacted with miR-543 and negatively regulated miR-543 expression in HeLa cells. Silencing of circRNA THBS1 remarkably suppressed HeLa cells’ viability, accelerated cells’ apoptosis, and inhibited the EMT of HeLa cells, while these changes were reversed by miR-543 inhibitor. Moreover, miR-543 affected HeLa cells by targeting HMGB2. In conclusion, circRNA THBS1 silencing inhibited the malignant biological behaviors of cervical cancer cells via the regulation of miR-543/HMGB2 axis.

## 1 Introduction

Cervical cancer is the most common gynecological malignancy, which has become a major health problem for women. Women with early marriage, early childbirth, fertility, and sexual disorders have a higher prevalence rate [1,2]. At present, surgery, chemotherapy, radiotherapy, and targeted therapy were applied for cervical cancer treatment [3,4]. However, the cure rate of middle- and late-stage cervical cancer patients is very low. Therefore, the basic research on the pathogenesis of cervical cancer is of great significance to improve the level of clinical treatment. In particular, it is necessary to explore the biological functions and regulatory effects of cervical cancer-related genes, study the molecular mechanisms related to the occurrence and development of cervical cancer, and seek therapeutic targets for cervical cancer.

Circular RNA (circRNA) is a new type of non-coding RNA molecule with closed circular structure [5]. Increasing reports have confirmed that circRNA plays an important regulatory role in the occurrence, development, and metastasis of tumors [6,7]. Therefore, cyclic RNA has the potential to become a biomarker for tumor diagnosis and a new target for treatment. A study has shown that circRNA THBS1 promotes the migration and invasion of non-small-cell lung cancer cells by adsorbing the expression of miR-129-5p regulating gene SOX4 [8]. However, the role and mechanism of circRNA THBS1 in cervical cancer is still unclear. It is necessary to further study the effect of circRNA THBS1 in cervical cancer cells and analyze the potential molecular regulation mechanism.

microRNA (miRNA) is a kind of endogenous non-coding RNA with a length of about 19–25 nt, which is widely involved in post-transcriptional regulation of genes [9]. Recent studies have shown that miRNA is involved in a variety of regulatory pathways, including organ formation, cell proliferation and apoptosis, fat metabolism, and so on, and plays an important role in gene expression regulation [10,11]. In various cancers, miRNA may act as a carcinogen or tumor suppressor to regulate tumor formation. miR-543 has been shown to promote cell proliferation and metastasis of renal cell carcinoma through Wnt/β-Catenin signaling pathway [12]. Besides, miR-543 promotes the proliferation and metastasis of colorectal cancer by targeting KLF4 [13]. Moreover, it was found that miR-543 was significantly downregulated in cervical cancer, and it played an anti-cancer role in cervical cancer [14]. Therefore, we hypothesized that circRNA THBS1 may play a role in cervical cancer by regulating the expression of miR-543.

Thus, our investigation aimed to explain the underlying functions of circRNA THBS1 and miR-543 in cervical cancer and elucidate latent mechanisms.

## 2 Materials and methods

### 2.1 Clinical specimen collection

Thirty pairs of cancerous tissues and adjacent normal tissues from patients with cervical cancer were collected at the Shanghai Mengchao Cancer Hospital. All specimens were rapidly frozen and stored in liquid nitrogen and preserved at −80°C for further analysis. The research procedure was approved by the ethics committee of Shanghai Mengchao Cancer Hospital. We obtained the written informed consent of all patients, and all patients agree to use tissue in the study.

### 2.2 Cell culture

Normal human cervical epithelial cell line (Ect1/E6E7) and cervical cancer cell lines (C-33A, SiHa, CaSki, and HeLa) were purchased from ATCC. The cells were cultivated in RPMI-1640 medium (Procell) containing 15% FBS (Procell) and 1% penicillin/streptomycin (Procell) in a humidified incubator containing 5% CO_2_ at 37°C.

### 2.3 Dual-luciferase reporter assay

starBase was used to identify the relationship between circRNA THBS1 and miR-543. The results indicated that miR-543 was the potential target of circRNA THBS1. The fragment of the circRNA THBS1 containing the miR-543-binding site or mutate target site was amplified by RT-PCR and then cloned into a pmirGLO vector (Promega, USA) to generate the reporter vector circRNA THBS1 wild type (circRNA THBS1-WT) or circRNA THBS1 mutated type (circRNA THBS1-MUT). circRNA THBS1-WT or circRNA THBS1-MUT and miR-543 mimics or mimic control were co-transfected into 293T cells using Lipofectamine 3000, respectively, and incubated for 48 h. Then, the relative luciferase activity was detected by Dual-Luciferase^®^ Reporter Assay System (Promega, USA) following the protocol. The relationship between miR-543 and HMGB2 was verified using the same method.

### 2.4 RT-qPCR analysis

After treatment, the levels of circRNA THBS1, miR-543, HMGB2, E-cadherin, and N-cadherin were measured by RT-qPCR. The isolation of RNA from cervical cancer tissue and cervical cancer cell lines (C-33A, SiHa, CaSki, and HeLa) was obtained with the TRIpure Total RNA Extraction Reagent (ELK Biotechnology) based on the protocol. Then, the total RNA was reversed to cDNA following the instructions of PrimeScript RT Reagent Kit (TaKaRa, China), and qPCR analysis was conducted using the SYBR PrimeScript RT-PCR Kit (TaKaRa) with ABI 7500 Real-Time PCR System (Agilent Technologies, USA). Target gene expressions were calculated using the 2^−ΔΔCt^ method.

### 2.5 Cell transfection

Control-siRNA, circRNA THBS1-siRNA, control plasmid, circRNA THBS1 plasmid, inhibitor control, miR-543 inhibitor, mimic control, miR-543 mimic, control plasmid, or HMGB2 plasmid were transfected into HeLa cells by Lipofectamine 3000 (Invitrogen) for 48 h referring to the instructions. After 48 h transfection, RNA was extracted for RT-qPCR analysis, and western blot was adapted to evaluate the protein expression.

### 2.6 MTT assay

After treatment, HeLa cells were implanted into 96-well plates and treated with 10 μl MTT solution and continuously incubated for further 4 h. Then, the supernatant was discarded and 100 μl of DMSO was added to dissolve lysate. Finally, the optical density at the wavelength of 570 nm was measured by a microplate reader (BIOTEK, USA) following the protocol.

### 2.7 Flow cytometry (FCM) assay

After digesting the cells with trypsin without EDTA, the HeLa cells were collected by centrifugation at 4°C for 5 min. After that, the cells were washed twice with pre-cooled PBS. Then 1× binding buffer was used to prepare cell suspension. For cell apoptosis assay, cells were assessed using the Annexin-V/propidium iodide Apoptosis Detection Kit (Beyotime) according to the manufacturer’s protocol. Finally, apoptotic cells were checked using a Flow cytometer (BD Biosciences) and analyzed with Kaluza Analysis software (version 2.1.1.20653; Beckman Coulter, Inc.).

### 2.8 Western blot assay

The HeLa cells were lysed using RIPA buffer (Beyotime) for 30 min on ice. Proteins were resolved by sodium dodecyl sulfate-polyacrylamide gel electrophoresis and then transferred onto polyvinylidene fluoride membranes. The membranes were blocked with 5% skimmed milk for 2 h to avoid nonspecific binding and then cultivated with primary antibodies against E-cadherin, N-cadherin, or GAPDH (1:1,000 dilution) at 4°C overnight. After washing in TBST, the membranes were cultivated with secondary antibodies for 2 h. The protein signals were assessed by electrogenerated chemiluminescence method following the instructions.

### 2.9 Statistical analysis

All the above experiments were performed three times. All results were presented as the mean ± standard deviation and analyzed by using GraphPad Prism 6.0. Differences among groups were estimated using one-way analysis of variance and Student’s *t*-test. *P < 0.05, and **P < 0.01 indicated statistically as significant difference.

## 3 Results

### 3.1 MiR-543 directly interacted with circRNA THBS1

**Figure 1:**
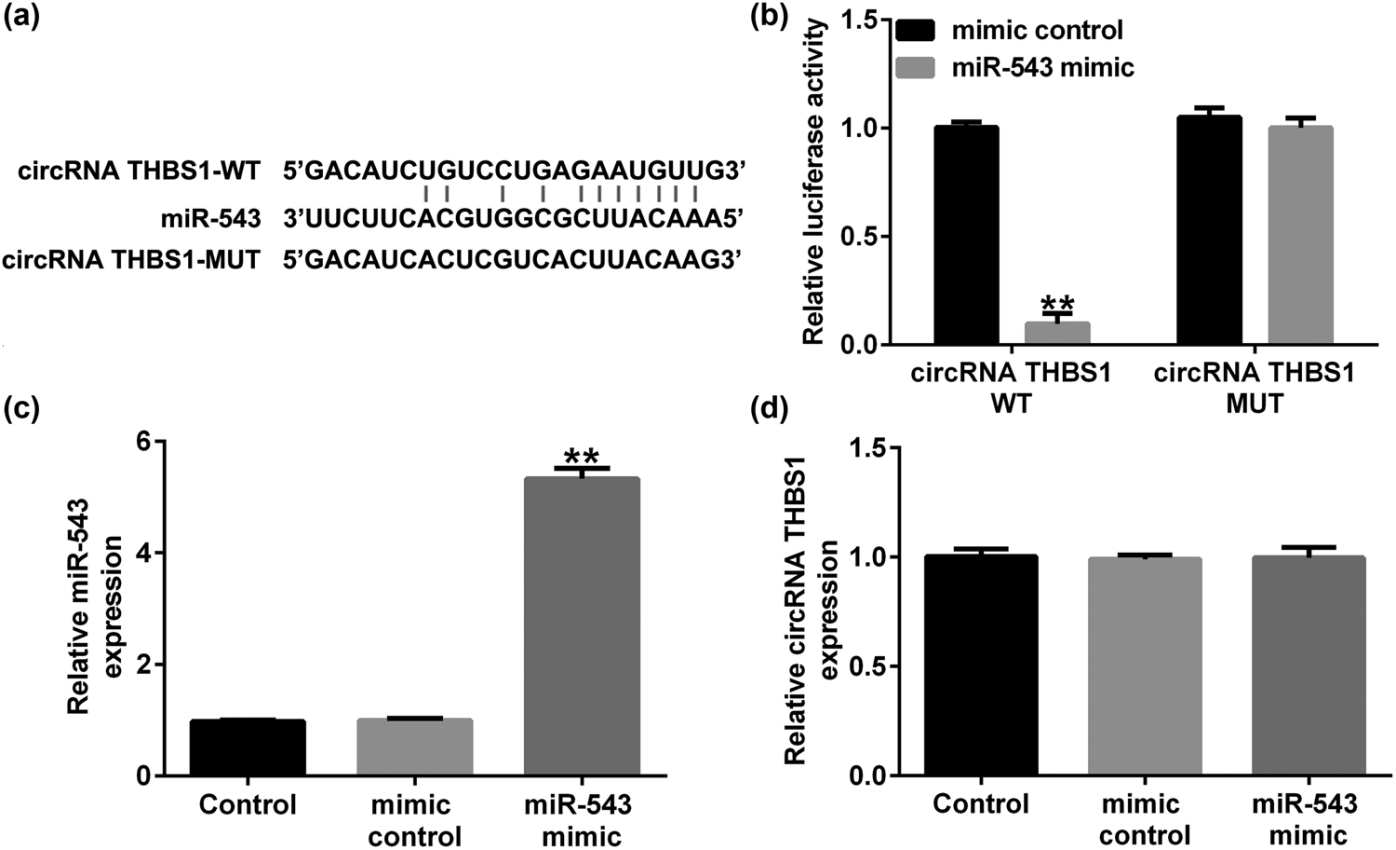
circRNA THBS1 sponged to miR-543. (a) The predicted binding site for miR-543 in the 3′-UTR of circRNA THBS1. (b) Luciferase activities were evaluated using Dual luciferase reporter assay in 293T cells. (c) Relative expression level of miR-543 in miR-543 mimic-transfected HeLa cells. (d) Relative expression level of circRNA THBS1 in miR-543 mimic-transfected cells. **P < 0.01 vs mimic control.

**Figure A1:**
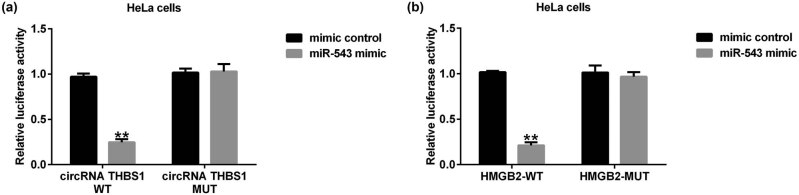
Dual-luciferase reporter assay in HeLa cells. (a) To verify the binding sites between miR-543 and circRNA THBS1, dual luciferase reporter assay was performed in HeLa cells. (b) To confirm the binding sites between miR-543 and HMGB2, dual luciferase reporter assay was performed in HeLa cells. **P<0.01 vs. mimic control.

We first explored the relevance between circRNA THBS1 and miRNAs, and the bioinformatics analysis (starBase) suggested that circRNA THBS1 harbored a binding site for miR-543 (Figure 1a). Dual-luciferase reporter assay further evidenced that miR-543 mimic remarkably reduced the relative luciferase activity of circRNA THBS1-WT, while the luciferase activity of circRNA THBS1-MUT was unaffected (Figure 1b). We also performed the dual luciferase reporter assay in HeLa cells, and similar results were obtained (Figure A1a). Moreover, we illustrated the effects of miR-543 on circRNA THBS1 in HeLa cells, and HeLa cells were transfected with mimic control and miR-543 mimic. The data indicated that compared with the mimic control group, miR-543 significantly enhanced miR-543 expression in HeLa cells (Figure 1c). However, miR-543 mimic had no significat effects on circRNA THBS1 expression in HeLa cells (Figure 1d). These findings demonstrated that circRNA THBS1 sponged miR-543 in the progression of cervical cancer.

### 3.2 CircRNA THBS1 was significantly upregulated and miR-543 was low-expressed in cervical cancer patient tissues and cervical cancer cell lines

**Figure 2:**
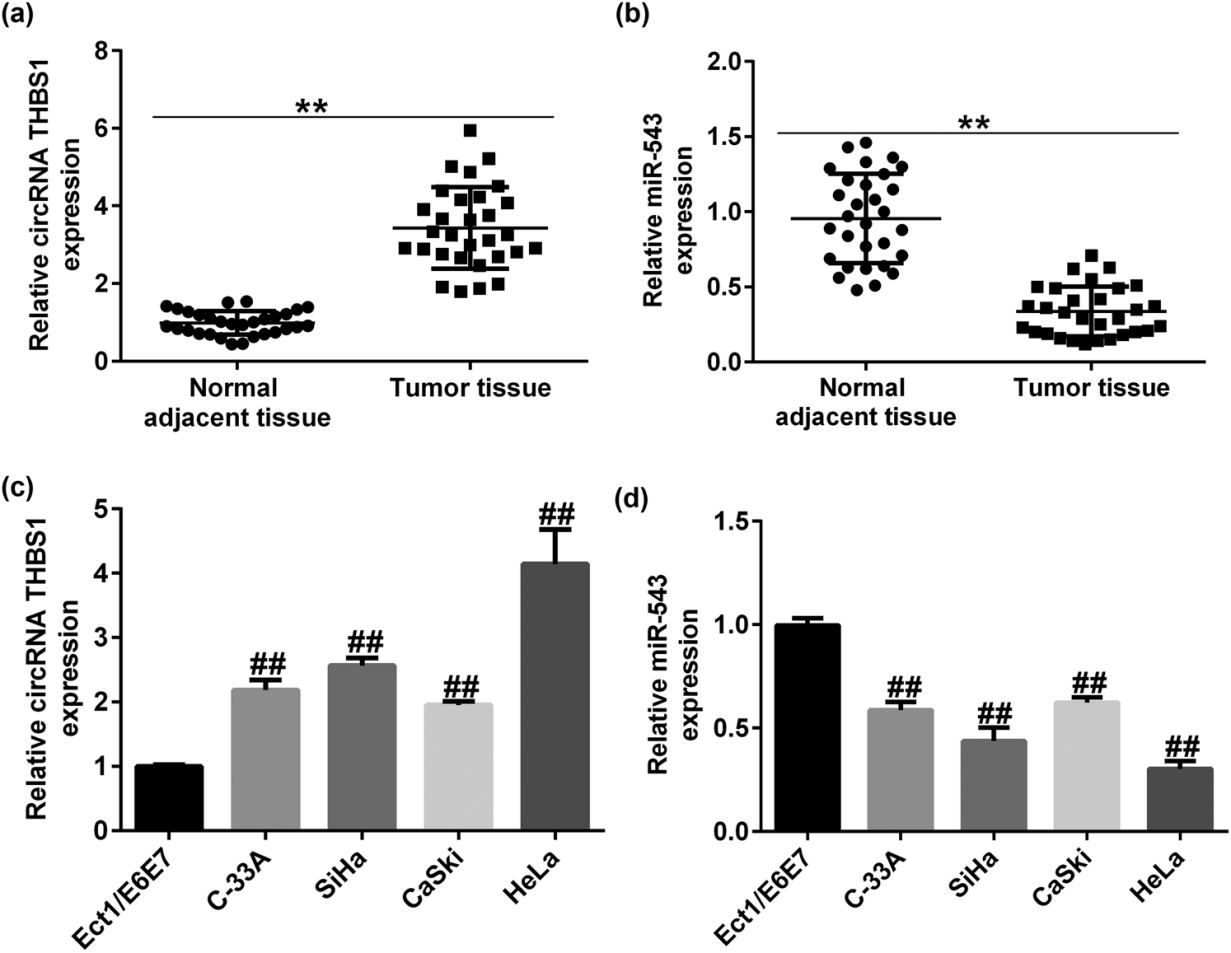
Expression of circRNA THBS1 and miR-543 in cervical cancer tissues and cells. RT-qPCR analysis of circRNA THBS1 (a) and miR-543 (b) expression in cervical cancer tissues and adjacent normal tissues. (c and d) Detection of circRNA THBS1 and miR-543 in cervical cancer cell line (C-33A, SiHa, CaSki, and HeLa) and Ect1/E6E7 cells. **P < 0.01 vs adjacent normal tissue; ^##^ *P* < 0.01 vs Ect1/E6E7.

We then analyzed the circRNA THBS1 and miR-543 expression in cervical cancer tissues and cell lines. RT-qPCR analysis revealed that circRNA THBS1 was upregulated and miR-543 was downregulated in cervical cancer patient tissues (Figure 2a and b), compared to normal paracancer tissues. As displayed in Figure 2c, the level of circRNA THBS1 was obviously higher in cervical cancer cell lines than that in Ect1/E6E7 cells. Besides, we observed that miR-543 was downexpressed in cervical cancer cell lines, as opposed to Ect1/E6E7 cells (Figure 2d). Our findings suggested that circRNA THBS1/miR-543 may play a regulatory role in cervical cancer. Due to the more significant difference in the expression of circRNA THBS1/miR-543 in the cervical cancer cell line HeLa, we thus choose HeLa cells for further research.

### 3.3 CircRNA THBS1 negatively regulated miR-543 expression in cervical cancer cell line HeLa

**Figure 3:**
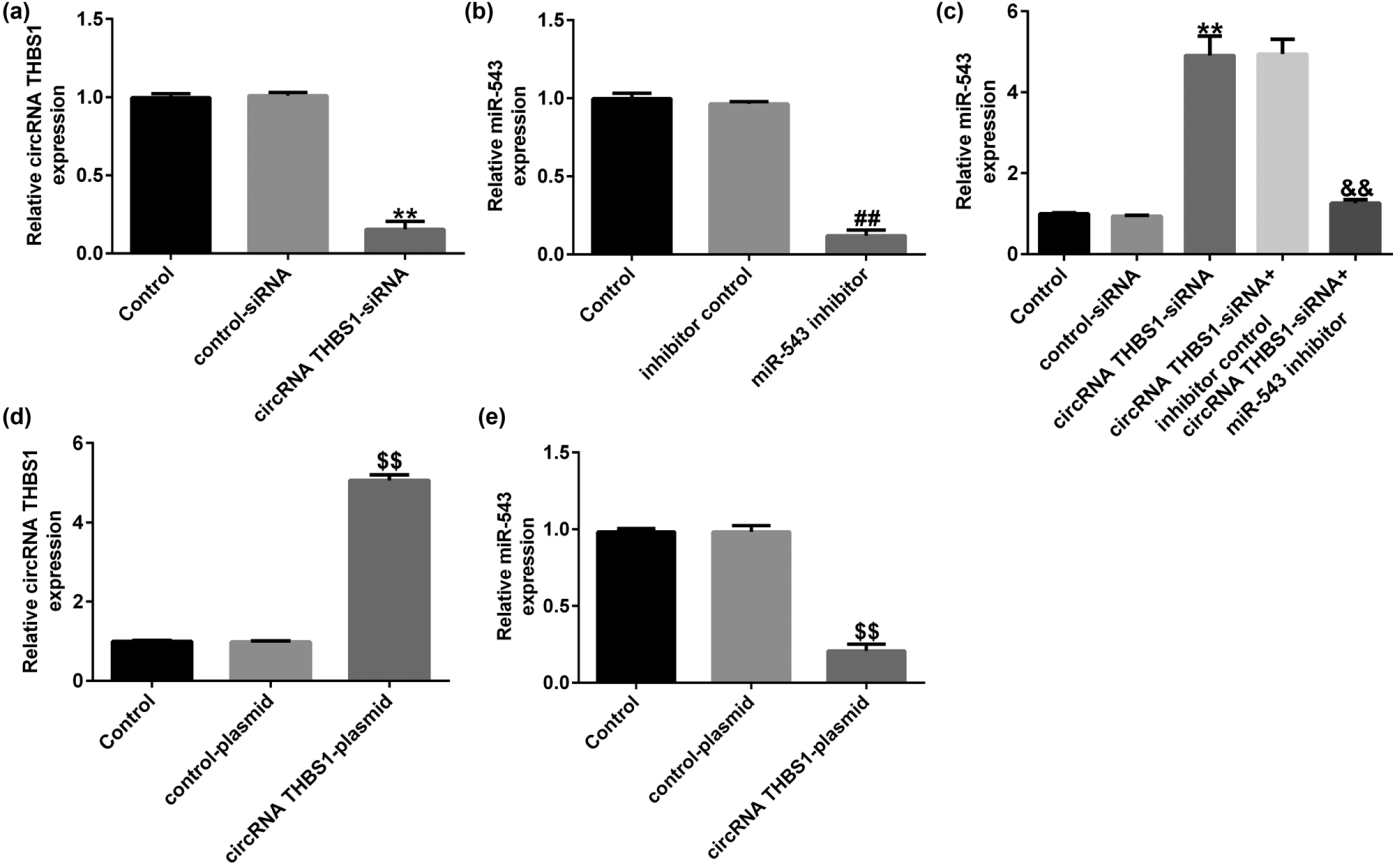
miR-543 inhibitor abolished the effects of circRNA THBS1 on miR-543 level in HeLa cells. (a) RT-qPCR analysis of circRNA THBS1 in control-siRNA or circRNA THBS1-siRNA-transfected HeLa cells. (b) mRNA levels of miR-543 in HeLa cells were detected by RT-qPCR. (c) miR-543 levels in control-siRNA + miR-543 inhibitor- or circRNA THBS1-siRNA + miR-543 inhibitor-transfected cells. (d) RT-qPCR analysis of circRNA THBS1 in control plasmid- or circRNA THBS1 plasmid-transfected HeLa cells. (e) RT-qPCR analysis of miR-543 in control plasmid- or circRNA THBS1 plasmid-transfected HeLa cells. **P < 0.01 vs control-siRNA; ^##^ *P* < 0.01 vs inhibitor control; ^&&^ *P* < 0.01 vs circRNA THBS1-siRNA + inhibitor control.

To further explain the regulatory roles of circRNA THBS1 and miR-543 in cervical cancer cells, control-siRNA, circRNA THBS1-siRNA, inhibitor control, or miR-543 inhibitor were transfected into HeLa cells for 48 h. RT-qPCR assay was applied to detect circRNA THBS1 and miR-543 levels in different groups. Our data revealed that circRNA THBS1-siRNA remarkably decreased circRNA THBS1 levels, compared to the control-siRNA group (Figure 3a). Furthermore, as displayed in Figure 3b, miR-543 was downregulated in miR-543 inhibitor-transfected HeLa cells, as opposed to the inhibitor control group. We also observed that circRNA THBS1-siRNA dramatically increased miR-543 expression in HeLa cells, while this promotion was abolished after miR-543 inhibitor treatment (Figure 3c). In addition, we found that compared with the control plasmid group, circRNA THBS1 plasmid significantly enhanced circRNA THBS1 expression in HeLa cells (Figure 3d), while miR-543 expression significantly reduced (Figure 3e). Based on these findings, we revealed that circRNA THBS1 negatively regulated miR-543 levels in cervical cancer cells.

### 3.4 Inhibition of miR-543 reversed the influence of circRNA THBS1-siRNA on HeLa cells viability, apoptosis, and EMT

**Figure 4:**
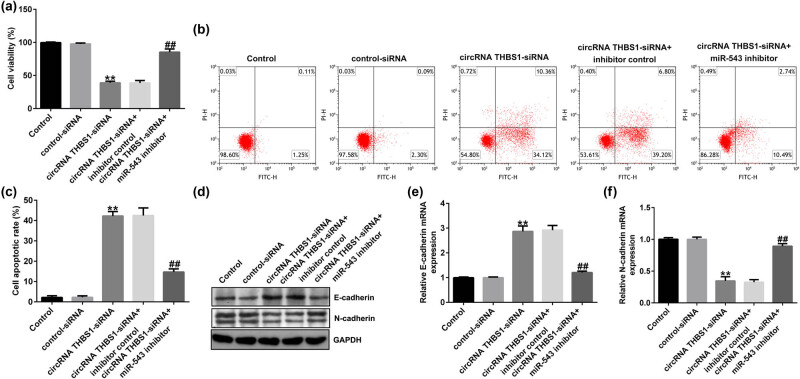
circRNA THBS1-siRNA suppressed HeLa cells’ viability, promoted cells’ apoptosis, and inhibited EMT by sponging to miR-543. (a) Cells’ viability and (b) cell apoptosis were determined by MTT and FCM analyses. (c) Quantification of apoptotic cells. (d) Western blot analysis of E-cadherin and N-cadherin expressions. mRNA levels of E-cadherin (e) and N-cadherin (f) were checked using RT-qPCR. **P < 0.01 vs control-siRNA; ^##^ *P* < 0.01 vs circRNA THBS1-siRNA + inhibitor control.

To better understand the functions of circRNA THBS1 or miR-543 in HeLa cells, HeLa cells were transfected with control-siRNA, circRNA THBS1-siRNA, circRNA THBS1-siRNA + inhibitor control, or circRNA THBS1-siRNA + miR-543 inhibitor for 48 h. Results from MTT and FCM assay demonstrated that circRNA THBS1-siRNA reduced HeLa cells’ viability (Figure 4a) and promoted more apoptotic cells (Figure 4b and c). Besides, our data revealed that circRNA THBS1-siRNA inhibited the EMT of cervical cancer cells, as confirmed by enhanced E-cadherin expression (Figure 4d and e) and suppressed N-cadherin level (Figure 4d–f). However, all these findings were reversed by miR-543 inhibitor co-transfection, suggesting that circRNA THBS1 was involved in the HeLa cells’ viability, apoptosis, and EMT by sponging to miR-543.

### 3.5 miR-543 directly targets HMGB2

**Figure 5:**
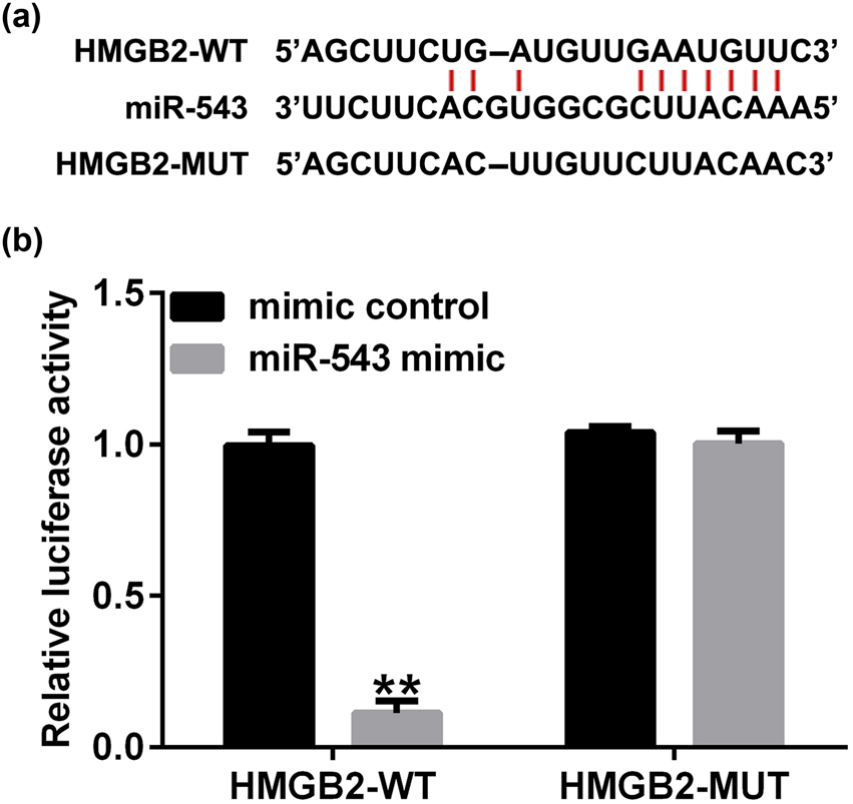
HMGB2 was a direct target of miR-543. (a) A schematic of miR-543 binding site in HMGB2 3′-UTR. (b) Dual-luciferase reporter assay system was applied for verifying the relationship between HMGB2 and miR-543. **P < 0.01 vs mimic control.

Having illustrated the correlation between circRNA THBS1 and miR-543, we further investigated the potential mechanisms of miR-543 in HeLa cells. According to bioinformatics tools (starBase), HMGB2 was a candidate target of miR-543 (Figure 5a). In addition, dual luciferase reporter gene system further suggested that HMGB2 was a direct target of miR-543 (Figure 5b). The dual luciferase reporter assay was also conducted in HeLa cells, and similar results were obtained (Figure A1b). All these observations above indicated that HMGB2 was a direct target of miR-543.

### 3.6 miR-543 negatively regulated HMGB2 expression in HeLa cells

**Figure 6:**
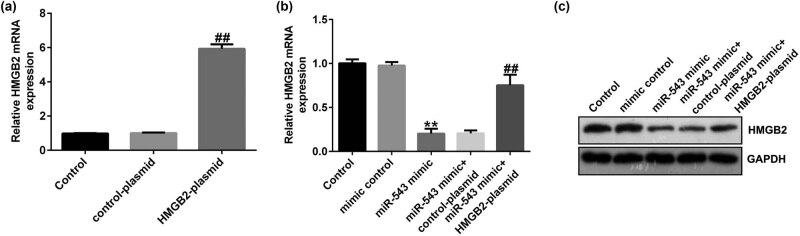
HMGB2 plasmid abolished the effects of miR-543 mimic HMGB2 level. (a) RT-qPCR analysis of HMGB2 in HeLa cells after HMGB2 plasmid or control plasmid transfection. (b) mRNA levels of HMGB2 in mimic control + HMGB2 plasmid- or miR-543 mimic + HMGB2 plasmid-transfected cells. (c) protein levels of HMGB2 in mimic control + HMGB2 plasmid- or miR-543 mimic + HMGB2 plasmid-transfected cells. **P < 0.01 vs mimic control; ^##^ *P* < 0.01 vs control plasmid; ^&&^ *P* < 0.01 vs miR-543 mimic + control plasmid.

We also illustrated the relationship between miR-543 and HMGB2 in HeLa cells, and HeLa cells were transfected with mimic control, miR-543 mimic, control plasmid, or HMGB2 plasmid for 48 h. Results from Figure 6a revealed that HMGB2 was markedly upregulated in HMGB2 plasmid-transfected cells, in comparison to the control plasmid group. Moreover, miR-543 mimic significantly reduced HMGB2 level in HeLa cells, while this inhibition was reversed after HMGB2 plasmid treatment (Figure 6b and c). According to these observations, we verified that miR-543 negatively regulated HMGB2 expression in HeLa cells.

### 3.7 Upregulation of miR-543 inhibited HeLa cells viability, EMT, and accelerated cells apoptosis by targeting HMGB2

**Figure 7:**
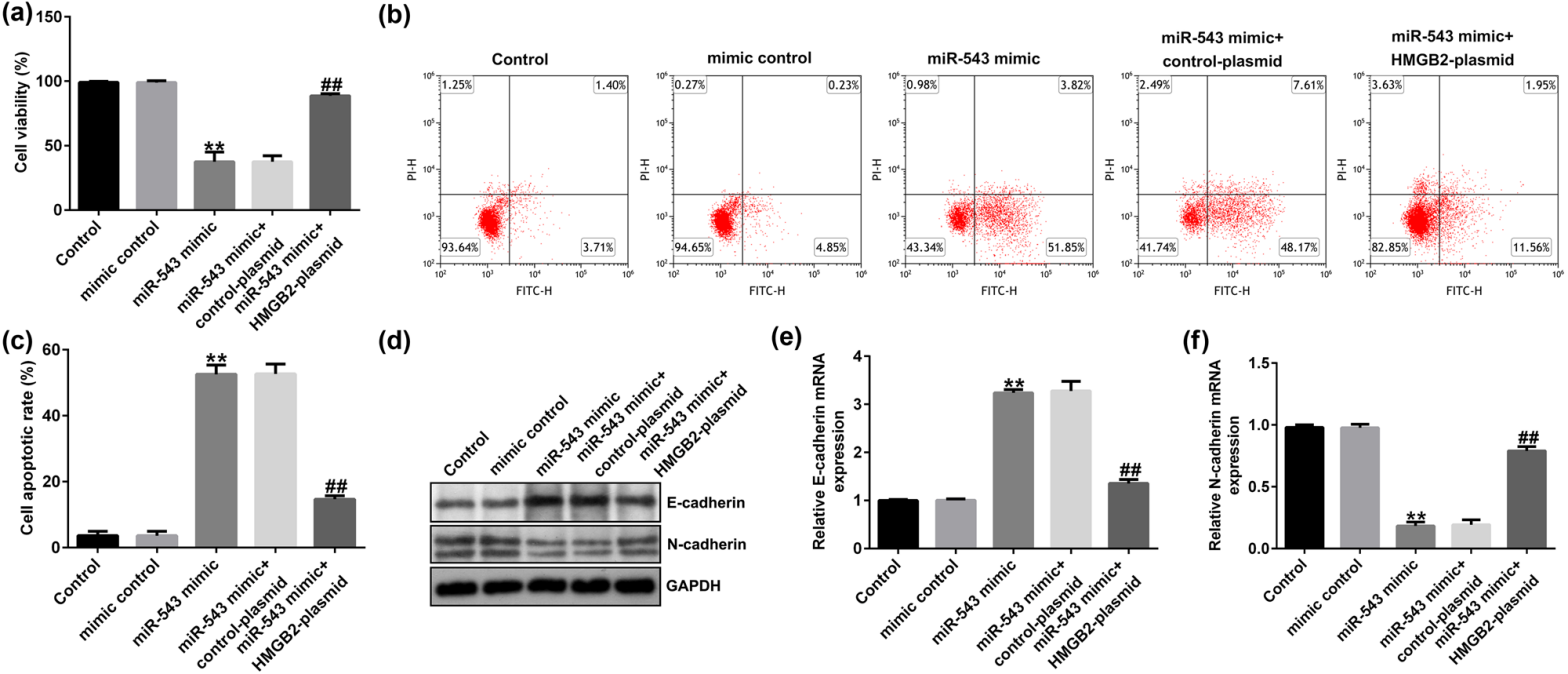
miR-543 mimic inhibited the biological behavior of cervical cancer cells via HMGB2. (a and b) Cells’ viability and apoptotic cells were checked using MTT and FCM analysis. (c) Quantitation of apoptotic cells. (d) Detection of E-cadherin and N-cadherin expression using Western blot analysis. (e and f) RT-qPCR analysis of E-cadherin and N-cadherin levels in miR-543 mimic + control plasmid- or miR-543 mimic + HMGB2 plasmid-transfected HeLa cells. **P < 0.01 vs mimic control; ^##^ *P* < 0.01 vs miR-543 mimic + control plasmid.

Finally, we explained the mechanism by which miR-543 mediated HeLa cells’ biological functions. Findings from Figure 7a and b revealed that miR-543 mimic suppressed HeLa cells’ viability and accelerated more apoptotic cells. Moreover, the expression of E-cadherin (Figure 7d and e) was increased and N-cadherin was reduced (Figure 7d and f) by miR-543 mimic, as opposed to the mimic control group. Nevertheless, all these findings were reversed by HMGB2 plasmid. The findings indicated that knockdown of circRNA THBS1 inhibited the malignant biological behavior of cervical cancer cells by miR-543/HMGB2 axis.

## 4 Discussion

The findings of the current study indicated that circRNA THBS1 was significantly upregulated and miR-543 was low-expressed in cervical cancer tissues and cell lines. circRNA THBS1 silencing inhibited cervical cancer cell proliferation and EMT and induced cell apoptosis via the regulation of miR-543/HMGB2 axis, suggesting its carcinogenic role in cervical cancer.

Cervical cancer, one of the most frequent malignant tumors in women, has become the main health problem for women due to its high morbidity and mortality [15,16]. At present, radical hysterectomy [17], chemoradiation [18], and immunotherapy [19] were applied for cervical cancer treatment. However, less than 20% of cervical cancer patients are eligible for surgery after diagnosis, indicating the significance of the early diagnosis of cervical cancer. Hence, increasing numbers of investigations focused on exploring new biomarkers, which may be applied for the diagnosis and treatment of cervical cancer.

circRNAs have been evidenced to be vital regulators in many diseases, including cervical cancer [20]. In addition, numerous studies have revealed that circRNAs participate in the biological behaviors of cervical cancer cells, including cell proliferation, apoptosis, and metastasis. For instance, Huang et al. evidenced that circ-ACACA promotes cervical cancer cells’ proliferation, invasion, and migration by regulating miR-582-5p/ERO1A signaling axis [21]. circRNA THBS1, a newly discovered circRNA, has been confirmed to regulate non-small-cell lung cancer cells’ functions by sponging miR-129-5p and regulating SOX4 expression [8]. Nevertheless, little is known about circRNA THBS1’s mechanism in cervical cancer development. First, we searched the underlying targets of circRNA THBS1 in cervical cancer, and the data suggested that circRNA THBS1 sponged to miR-543, suggesting a possible relationship of circRNA THBS1 and miR-543 in cervical cancer. We also evaluated the levels of circRNA THBS1 and miR-543 in cervical cancer tissues and cervical cancer cell lines, and the data indicated that circRNA THBS1 was significantly upregulated and miR-543 was downregulated in cervical cancer tissues and cervical cancer cell lines. Our data suggested that circRNA THBS1 and miR-543 were dysregulated in cervical cancer. miR-543 was found to act as oncogene or tumor suppressor in various cancers, including glioma [22], non-small-cell lung cancer [23], and colorectal cancer [13]. However, the biological functions of miR-543 in cervical cancer are largely indistinct. Thus, we speculated that circRNA THBS1 downregulation or miR-543 upregulation may prevent tumorigenesis in cervical cancer.

The etiology of cancer is highly complex, and it has several biological behaviors including cell invasion, neo-angiogenesis, EMT, and abnormal cell proliferation and apoptosis [24–26]. Besides, accumulating evidence suggests that immune cells, adhesion molecules, extracellular matrix metalloproteinase, and pro-inflammatory cytokines activate/alter microenvironment, creating the conditions for differentiation, adhesion, proliferation, and survival of cancer cells [27,28]. In this study, we explored the role of circRNA THBS1/miR-543 in HeLa cell proliferation, apoptosis, and EMT. We found that circRNA THBS1 negatively regulated miR-543 levels in cervical cancer cells. circRNA THBS1-siRNA significantly reduced HeLa cells’ viability and EMT and promoted cell apoptosis. However, all these findings were reversed by miR-543 inhibitor, suggesting that silencing of circRNA THBS1 had available proliferation and EMT inhibition, and apoptosis promotion effect in cervical cancer cells.

Evidence has verified that dysregulation of miRNAs is associated with their biological functions by targeting mRNAs in cervical cancer. Han et al. suggested that miR-99a inhibited cervical cancer cells’ proliferation and migration by targeting IGF1R [29]. We further investigated the potential mechanisms of miR-543 in HeLa cells. The findings indicated that miR-543 directly targets HMGB2, and it negatively regulated HMGB2 expression in HeLa cells. Liu et al. suggested that miR-543 inhibited cells’ growth and metastasis by targeting TRPM7 in cervical cancer [14]. Similar results were observed in our research, and the results demonstrated that miR-543 mimic significantly reduced cell viability, promoted cell apoptosis, and inhibited EMT in HeLa cells by targeting HMGB2.

## 5 Conclusion

Our findings strongly showed that circRNA THBS1 regulates cervical cancer cell proliferation, apoptosis, and EMT via regulating miR-543/HMGB2 axis. The findings may help to provide a prognostic biomarker and molecular therapeutic target for cervical cancer.
